# Arbidol HCl Inhibits PRRSV Infection by Downregulating CD163 and MYH9 Expression and Modulating Inflammatory Responses

**DOI:** 10.1155/tbed/2505057

**Published:** 2026-10-31

**Authors:** Li Chen, Mengxue Gao, Xiaoyang Zhan, Lei Kong, Shuzhen Jiang, Suimin Shang, En-xin Li, Xiaoting Peng, Qiuju Dai, Yubao Li, Yuyu Zhang, Muhammad Zahoor Khan, Liangliang Li, Jiaqiang Wu, Tongtong Wang

**Affiliations:** ^1^ College of Agriculture and Biology Liaocheng University Liaocheng China lcu.edu.cn; ^2^ Shandong Provincial General Station of Animal Husbandry Jinan 250100 China; ^3^ College of Animal Science and Technology Shandong Agricultural University Tai’an China sdau.edu.cn; ^4^ Key Laboratory of Livestock and Poultry Multi-Omics of MARA Institute of Animal Science and Veterinary Medicine Shandong Academy of Agricultural Sciences Jinan 250100 China saas.ac.cn

**Keywords:** adsorption and internalization, anti-PRRSV drug, Arbidol HCl, CD163 and MYH9 proteins, TLR4/NF-κB signaling pathway

## Abstract

Porcine reproductive and respiratory syndrome (PRRS) constitutes a major challenge for global pig production, yet effective therapeutic interventions remain elusive. Our study investigated the antiviral potency of Arbidol HCl in counteracting PRRSV infection through comprehensive in permissive cells and in vivo approaches. Our observations demonstrate that Arbidol HCl confers an obvious antiviral activity against varied PRRSV isolates in MARC‐145 cells and primary porcine alveolar macrophages (PAMs). Mechanistic studies revealed that Arbidol HCl inhibits the early stages of PRRSV infection by significantly downregulating the expression of key viral receptors cysteine‐rich scavenger receptor (CD163) and myosin heavy chain 9 (MYH9), thereby disrupting viral attachment and internalization. Subsequent validation studies indicated that Arbidol HCl attenuates proinflammatory responses caused by PRRSV via suppressing the toll‐like receptor 4 (TLR4)/nuclear factor kappa B (NF‐κB) signaling pathway. In vivo experiments using PRRSV‐infected piglets demonstrated that Arbidol HCl treatment significantly reduced viral loads in serum, alleviated lung pathological damage, and mitigated systemic inflammatory responses. These results collectively establish Arbidol HCl as a promising dual‐action therapeutic agent that combines direct antiviral effects with anti‐inflammatory properties. Our study offers hope for developing a practical therapeutic intervention that could significantly impact swine health and food security worldwide. It will provide a novel approach for PRRS prevention and treatment in swine production.

## 1. Introduction

Porcine reproductive and respiratory syndrome (PRRS) is a highly significant transboundary infectious disease affecting the swine industry, which was characterized by fever, anorexia, severe respiratory symptoms, reproductive disorders, and immunosuppression in pig [1–3]. As a positive‐sense, single‐stranded RNA virus possessing an envelope, PRRSV is classified within the Arteriviridae family under the order Nidovirales [4]. Since its discovery in the late 1980s, two genotypes of PRRSV, designated PRRSV‐1 and PRRSV‐2, have been identified worldwide [5]. Four distinct lineages of PRRSV‐2 (lineages 1, 3, 5, and 8) and PRRSV‐1 have been identified in different regions of China in recent years [6–8].

Due to the strong transmissibility, genetic heterogeneity of PRRSV, and both innate and adaptive immune responses, damage is induced by PRRSV in the host [9, 10]. Currently, available vaccines provide only limited cross‐protection against heterologous strains, and no specific antiviral therapeutics have been approved for clinical use in swine [11]. This significant gap in disease management underscores the urgent need to identify and develop potent antiviral therapeutic candidates against PRRSV.

Arbidol HCl displays diverse biological activities, including antibacterial, antiviral, anti‐inflammatory, anticancer, and antioxidant properties [12–14]. Arbidol HCl has demonstrated efficacy against several viral infections. For instance, Kadam and Wilson [15] elucidated that Arbidol HCl inhibits influenza virus infection by blocking viral fusion using structural biology approaches. Zhao et al. [16] reported that Arbidol HCl reduces Omicron severe acute respiratory syndrome coronavirus 2 (SARS‐CoV‐2) infection in patients. Additionally, accumulated evidence indicate that Arbidol HCl is capable of inhibiting herpes simplex virus 1 (HSV‐1) [17], HSV II (HSV‐2) [18], and respiratory syncytial virus (RSV) infection [19]. However, the effectiveness and precise mechanism of Arbidol HCl against PRRSV remain ambiguous.

We report here a systematic evaluation of Arbidol HCl as an anti‐PRRSV agent, carried out in cell culture and in piglets, together with the mechanism that underlies its activity. The compound curtailed PRRSV replication in MARC‐145 cells and in porcine alveolar macrophages (PAMs), acting at adsorption and internalization through the downregulation of CD163 and myosin heavy chain 9 (MYH9). It additionally restrained the excessive proinflammatory output provoked by infection, an effect attributable to interference with toll‐like receptor 4 (TLR4)/nuclear factor kappa B (NF‐κB) signaling. In challenged piglets, treatment lowered the circulating viral load and limited pulmonary lesions. Combining these two properties in a single molecule makes Arbidol HCl an attractive starting point for PRRS therapy.

## 2. Results

### 2.1. The Drug Cytotoxicity Detection of Arbidol HCl

Figure 1A depicts the structural formula of Arbidol HCl. Before antiviral activity was examined, the highest concentration tolerated by the two PRRSV‐permissive cell types was established. The viability of MARC‐145 cells and of PAMs was unaffected by exposure to the compound at up to 20 µM (Figure 1B), and this dose was therefore adopted as the ceiling for all subsequent work.

**Figure 1:**
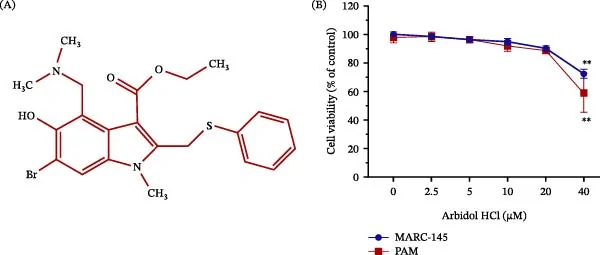
Chemical structure of Arbidol HCl and its tolerability in permissive cells. (A) Chemical structure of Arbidol HCl. (B) The MARC‐145 cells and PAMs at 90% confluence in 96‐well plates were exposed to Arbidol HCl at 0, 2.5, 5, 10, 20, or 40 μM for 24 h and viability measured with a CCK‐8 kit. Values come three separate experiments, with untreated wells taken as 100%. Comparisons against the 0 µM wells used Student’s *t*‐test;  ^∗∗^ *p* < 0.01.

### 2.2. Anti‐PRRSV Activity of Arbidol HCl in Susceptible Cells

To examine the antiviral effects of Arbidol HCl against PRRSV activity, MARC‐145 cells and PAMs were pretreated with varying doses of Arbidol HCl for 2 h, followed by infection with PRRSV SD16. Both culture supernatants and cell pellets were obtained to analyze the PRRSV replication using quantitative PCR (qPCR) and western blot. As shown in Figure 2A, compared with the dimethyl sulfoxide (DMSO)‐treated control group, progeny virus production decreased significantly in Arbidol HCl‐treated MARC‐145 cells with dose‐dependent characteristics. Correspondingly, PRRSV N protein expression was markedly reduced (Figure 2B). Similar anti‐PRRSV effects of Arbidol HCl were observed in PAM cells (Figure 2C,D). Moreover, immunofluorescence assay (IFA) results demonstrated that Arbidol HCl reduced PRRSV protein expression in a concentration‐dependent manner both on MARC‐145 cells and PAMs (Figure 2E).

**Figure 2:**
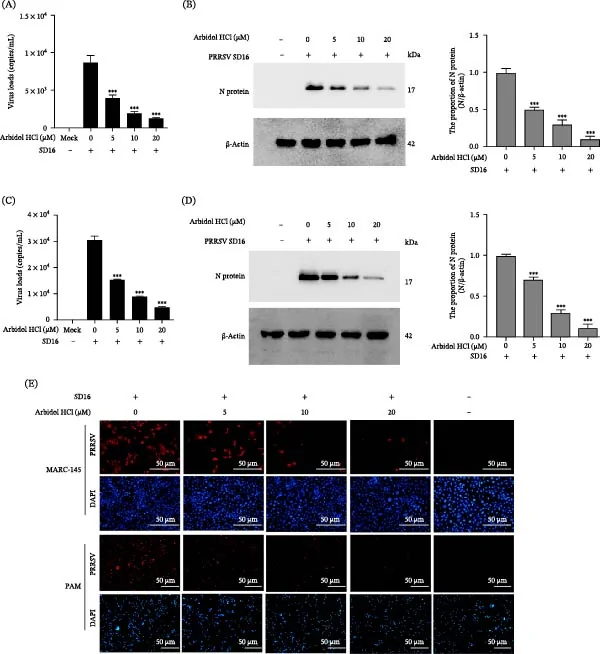
Arbidol HCl suppresses significantly PRRSV replication in permissive cells. MARC‐145 (A, B) and PAM (C, D) cultures were held with Arbidol HCl at 0, 5, 10, or 20 µM for 2 h, inoculated with PRRSV SD16 at 0.1 MOI for 1 h and then returned to 3% FBS DMEM containing the corresponding dose. Supernatants and cells taken at 24 hpi were examined for PRRSV replication by qPCR and western blot, with β‐actin as loading control. Three independent experiments were analyzed by one‐way ANOVA;  ^∗∗∗^ *p* < 0.001 against the 0 µM wells. (E) MARC‐145 cells and PAMs were pretreated with 0, 5, 10, or 20 µM Arbidol HCl were infected with, RRSV SD16 (MOI = 0.1) and kept in the corresponding dose. At 36 hpi the monolayers were fixed and stained with anti‐PRRSV serum (red) and DAPI (blue) and imaged on a Leica DMi8 fluorescence microscope. Scale bar, 50 µm.

### 2.3. Arbidol HCl Exerts a Broad‐Spectrum Antiviral Activity Against PRRSV Strains

To evaluate whether Arbidol HCl possesses antiviral efficacy against other PRRSV strains, we examined its effects on different PRRSV‐1 strains and PRRSV‐2 strains. The results of TCID_50_ and western blot showed that Arbidol HCl significantly reduced virus titer and N protein expression of PRRSV‐1 GZ11‐G1 and P073‐3 strains in MARC‐145 cells (Figure 3A,B) and PAMs (Figure 3C,D). Notably, Arbidol HCl markedly decreased the titer of progeny virus following infection with PRRSV‐2 strains VR‐2332, JXA1, CH‐1a, and NADC30‐like in both MARC‐145 cells and PAMs (Figure 3E,G). Consistent with the TCID_50_ results, N protein expression levels were significantly reduced in Arbidol HCl‐treated cells (Figure 3F,H). Collectively, these findings demonstrate that Arbidol HCl possesses broad‐spectrum anti‐PRRSV activity.

**Figure 3:**
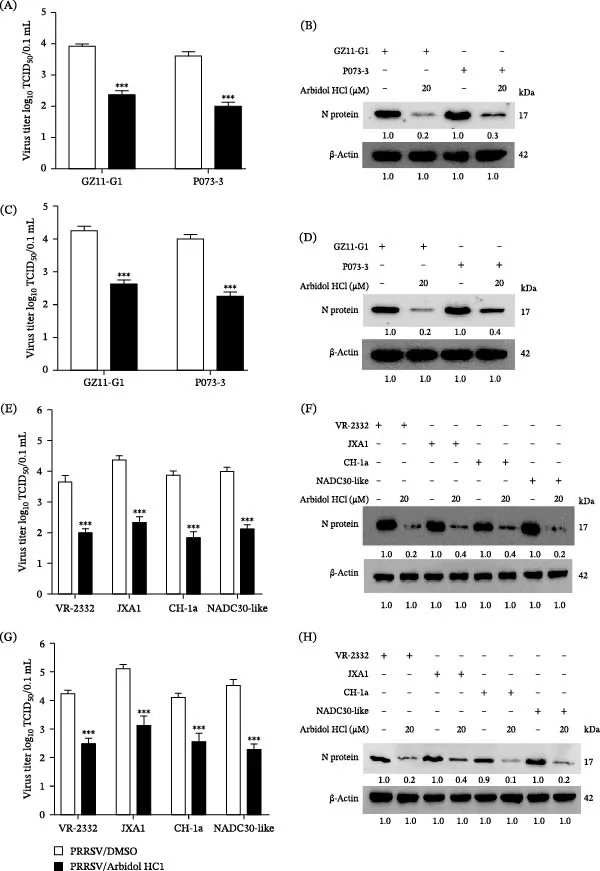
Arbidol HCl restricts both PRRSV‐1 and PRRSV‐2 strains in vitro. MARC‐145 cells (A, B) and PAMs (C, D) were held with 0 or 20 µM Arbidol HCl for 2 h and then inoculated for 1 h with the PRRSV‐1 isolates GZ11‐G1 or P073‐3 at 0.1 MOI. Supernatants and lysates taken at 24 hpi were assayed for virus titer and N protein by TCID_50_ and western blot. The same design was applied to MARC‐145 cells (E, F) and PAMs (G, H) challenged with the PRRSV‐2 isolates VR‐2332, JXA1, CH‐1a, and NADC30‐like. β‐Actin provided the loading control. Three independent replicates were compared by Student’s *t*‐test;  ^∗∗∗^ *p* < 0.001 against the DMSO‐treated group (0 µM Arbidol HCl).

### 2.4. Arbidol HCl Exerts Anti‐PRRSV Effects at the Early Infection Stages by Modulating CD163 and MYH9 Expression

A direct viral inactivation experiment was conducted in vitro to determine whether Arbidol HCl could directly inactivate PRRSV particles. As shown in Figure 4A, the number of progeny viral particles in the Arbidol HCl‐treated PRRSV infection group was not significantly different from that in the DMSO‐treated PRRSV infection group. Consistent results for N protein expression were observed between Arbidol HCl‐treated and DMSO‐treated PRRSV infection groups (Figure 4B). These data indicated that Arbidol HCl exerts no direct virucidal effect on PRRSV.

**Figure 4:**
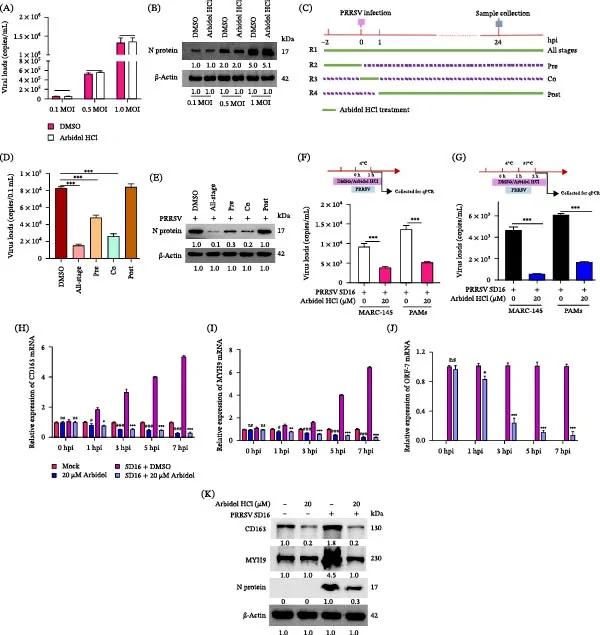
Arbidol HCl acts at the earliest steps of the PRRSV cycle. (A, B) PRRSV SD16 at 0.1, 0.5, or 1 MOI was held with 20 µM Arbidol HCl or DMSO for 2 h at 37°C before inoculation of MARC‐145 cells; progeny virus and N protein were measured at 24 hpi by qPCR (A) and western blot (B). (C) Design of the time‐of‐addition experiment, in which MARC‐145 cells received 20 µM Arbidol HCl at distinct time points before, during or after infection with PRRSV SD16 (MOI = 0.1), or throughout the cycle. Supernatants and cell pellets were examined for replication by qPCR (D), and western blot (E). (F) Attachment. MARC‐145 cells or PAMs held with Arbidol HCl (20 µM) or DMSO for 1 h at 37°C were washed, exposed to PRRSV SD16 at 1 MOI for 1 h at 4°C, and bound genomes counted by qPCR. (G) Internalization. After the same pretreatment and a 1 h binding step at 4°C with PRRSV SD16 (1 MOI), the cells were washed and returned to 37°C for a further 1 h with 20 µM Arbidol HCl or DMSO before genomes were counted by qPCR at 24 hpi. Values come from three independent replicates, are given as mean ± SD and were compared by Student’s *t*‐test;  ^∗∗∗^ *p* < 0.001 relative to DMSO‐treated cells (0 µM Arbidol HCl). (H–J) PAMs held with Arbidol HCl (20 µM) or DMSO for 2 h were infected with PRRSV SD16 at 0.1 MOI and sampled at 1, 3, 5, and 7 hpi for qPCR measurement of CD163 (H), MYH9 (I), and ORF‐7 (J). (K) Immunoblot of CD163 and MYH9 in mock, PRRSV SD16/DMSO and PRRSV SD16/Arbidol HCl cultures. GAPDH served as the internal control. The data shown from three independent replicates are shown. Data from three independent replicates are shown as mean ± SD and were compared by Student’s *t*‐test;  ^∗∗∗^ *p* < 0.001,  ^∗∗^ *p* < 0.01,  ^∗^ *p* < 0.05 against PRRSV + DMSO cells; ###*p* < 0.001, #*p* < 0.05 against the mock group.

Subsequently, a time‐of‐addition assay was carried out as depicted in Figure 4C,D to determine which stage of the PRRSV replication cycle is affected by Arbidol HCl. MARC‐145 cells were pretreated, cotreated, posttreated, or continuously treated with Arbidol HCl prior to PRRSV inoculation. Culture supernatants and cell fractions were harvested at 24 h postinfection (hpi) to quantify progeny virus production and N protein expression. As shown in Figure 4D,E, viral copy numbers and N protein expression were lower obviously in the Arbidol HCl all stage‐treated, pretreated, and cotreated groups compared to the DMSO‐treated control group. These data suggest that Arbidol HCl suppresses PRRSV infection during the early stages of viral infection.

Previous studies have shown that the PRRSV infectious cycle consists of four distinct stages: attachment, internalization, replication, and release [20, 21]. Therefore, the early stages of viral infection, including viral attachment and internalization, and qPCR, were performed to further determine whether the antiviral activity of Arbidol HCl is associated with reduced viral attachment or internalization in vitro. The results revealed that compared with the DMSO‐treated control group, Arbidol HCl treatment significantly decreased PRRSV copy numbers during the attachment stage in MARC‐145 cells (Figure 4F). A similar reduction of viral copy numbers was also observed during the internalization stage (Figure 4G). These findings indicate that Arbidol HCl inhibits PRRSV infection at both the attachment and entry stages.

Previous studies have reported that CD163 and MYH9 serve as cellular receptors or factors that play important roles in PRRSV entry into host cells [22, 23]. Next, we investigated whether the anti‐PRRSV activity of Arbidol HCl was associated with regulation of CD163 or MYH9 expression. PAMs were pretreated with Arbidol HCl (20 μM) for 2 h and subsequently infected with PRRSV SD16. Cells were collected at various time points to analyze the mRNA expression of CD163, MYH9, and ORF‐7 by qPCR. The results demonstrated that Arbidol HCl significantly decreased mRNA expression of CD163 (Figure 4H) and MYH9 (Figure 4I) in PAMs at 1, 3, 5, and 7 hpi; meanwhile, ORF‐7 expression reduced in a time‐dependent manner (Figure 4J). Notably, no significant differences were observed among the three groups at 0 h. We also examined CD163 and MYH9 protein expression in PAMs by western blot at 24 hpi. As shown in Figure 4K, compared with the DMSO‐treated group, Arbidol HCl markedly reduces CD163 and MYH9 expression in PAMs.

### 2.5. Arbidol HCl Attenuates Multiple Proinflammatory Cytokine Generation in PAMs

Previous studies have reported that upregulation of proinflammatory factors induced by PRRSV infection enhances viral pathogenicity both in vitro and in vivo [21, 24, 25]. To determine whether Arbidol HCl affects the expression of IL‐6, IL‐8, TNF‐α, and IL‐1β, PAMs were stimulated using PRRSV SD16 at 0.1 MOI for 24 h in the presence or absence of 10 or 20 μM Arbidol HCl. Culture supernatants from PRRSV‐infected PAMs were harvested at 24 hpi to analyze the IL‐6, IL‐8, IL‐1β, and TNF‐α levels using ELISA. The results showed that Arbidol HCl significantly decreased the production of IL‐6 (Figure 5A), IL‐8 (Figure 5B), IL‐1β (Figure 5C), and TNF‐α (Figure 5D) in a dose‐dependent manner. These findings indicate that Arbidol HCl can decrease different proinflammatory cytokine expression induced by PRRSV infection.

**Figure 5:**
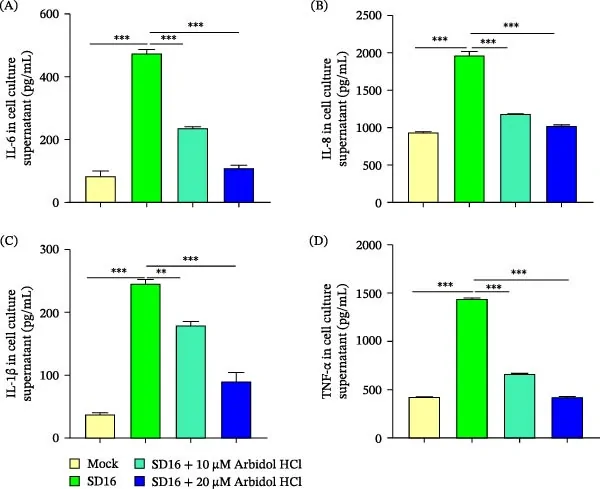
Arbidol HCl tempers the proinflammatory response of PAMs to PRRSV. PAMs were exposed to PRRSV SD16 at 0.1 MOI for 24 h with or without Arbidol HCl at 10 or 20 μM, and the culture fluids assayed for IL‐6 (A), IL‐8 (B), IL‐1β (C), and TNF‐α (D). Three separate experiments were compared by one‐way ANOVA;  ^∗∗^ *p* < 0.01 and  ^∗∗∗^ *p* < 0.001 against SD16‐infected cells.

### 2.6. Arbidol HCl Inhibits the TLR4/NF‐κB Signaling Pathway to Suppress PRRSV Infection

Previous studies have reported that the TLR4 and NF‐κB signaling pathways play pivotal roles in inflammatory and immune responses in mammalian cells [26, 27]. To further confirm whether the TLR4/NF‐κB pathway is involved in the anti‐PRRSV effect of Arbidol HCl, PAMs were pretreated with Arbidol HCl at different concentrations (5, 10, and 20 µM) for 2 h and subsequently infected with PRRSV SD16 at an MOI of 0.1. The expression of key proteins in the TLR4/NF‐κB pathway was detected by western blot. As shown in Figure 6A, following Arbidol HCl treatment, the expression levels of TLR4, MyD88, p‐NF‐κB, and p‐IκBα decreased in a dose‐dependent manner. Conversely, IκBα expression increased in a dose‐dependent characteristic, while total NF‐κB expression remained unchanged. These findings suggest that Arbidol HCl exerts its anti‐PRRSV effect by suppressing the TLR4/NF‐κB pathway.

**Figure 6:**
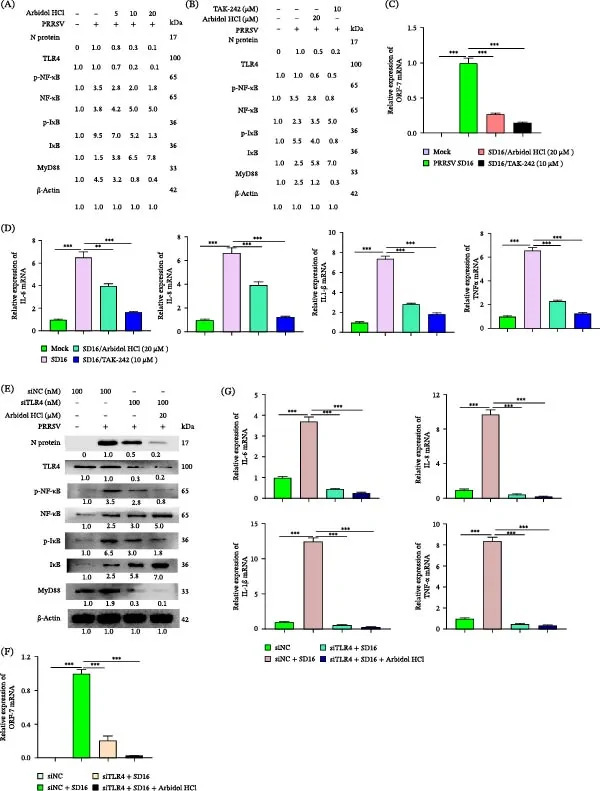
Arbidol HCl limits inflammation through suppression of TLR4/NF‐κB signaling. (A) PAMs held with Arbidol HCl at 5, 10, or 20 µM for 2 h were infected with PRRSV SD16 (MOI = 0.1) for 1 h and, 24 h later, immunoblotted for N protein, TLR4, p‐NF‐κB, NF‐κB, p‐IκBα, IκBα, and MyD88. (B) PAMs pretreated for 2 h with Arbidol HCl (20 µM) or the TLR4 inhibitor TAK‐242 (10 µM) were infected with PRRSV SD16 (0.1 MOI) and probed for the same panel at 24 hpi. (C, D) PRRSV ORF‐7 (C) together with IL‐6, IL‐8, IL‐1β, and TNF‐α (D) were quantified by qPCR in the same samples. (E) PAMs were transfected with siTLR4 for 12 h were infected with PRRSV SD16 (0.1 MOI) for 24 h in the presence or absence of Arbidol HCl (20 µM) and immunoblotted for the panel above, band intensities being measured in ImageJ. (F, G) PRRSV ORF‐7 (F) and IL‐6, IL‐8, IL‐1β, and TNF‐α (G) were quantified by qPCR. GAPDH served as the internal control. Values from at least three independent experiments are given as mean ± SD and were compared by Student’s *t*‐test;  ^∗∗^ *p* < 0.01 and  ^∗∗∗^ *p* < 0.001 against DMSO + SD16 treated group or the siNC + SD16 treated group.

Furthermore, PAMs were pretreated with either Arbidol HCl or TAK‐242 (a specific TLR4 inhibitor) and subsequently infected with PRRSV SD16. The expression of PRRSV N protein, TLR4, MyD88, p‐NF‐κB, NF‐κB, p‐IκBα, and IκBα was detected by western blot. As expected, PRRSV N protein expression at both the mRNA and protein levels in the TAK‐242‐treated group was similarly reduced as in the Arbidol HCl‐treated group. Consistent protein expression patterns of TLR4, MyD88, p‐NF‐κB, NF‐κB, p‐IκBα, and IκBα were observed between the TAK‐242‐treated and Arbidol HCl‐treated groups (Figure 6B,C). Notably, the expression levels of IL‐6, IL‐8, IL‐1β, and TNF‐α showed similar downward trends in both the TAK‐242‐treated and Arbidol HCl‐treated groups (Figure 6D).

Additionally, PAMs were transfected with either siTLR4 or siNC, treated with 20 µM Arbidol HCl, and then infected with PRRSV SD16. All cells were harvested to analyze PRRSV N protein, TLR4, MyD88, p‐NF‐κB, NF‐κB, p‐IκBα, and IκBα expression. As depicted in Figure 6E,F, compared with the siNC‐treated group, siTLR4 transfection markedly decreased the protein expression of TLR4, MyD88, p‐NF‐κB, and p‐IκBα, resulting in reduced N protein expression. Meanwhile, siTLR4 transfection also reduced the expression of IL‐6, IL‐8, IL‐1β, and TNF‐α (Figure 6G). These data suggest that the antiviral activity of Arbidol HCl is dependent on downregulation of the TLR4/NF‐κB signaling pathway.

### 2.7. Arbidol HCl Demonstrates Anti‐PRRSV Activity In Vivo

The protective effect of Arbidol HCl in piglets was further evaluated as illustrated in Figure 7A. Following administration of Arbidol HCl or DMSO, piglets were challenged with PRRSV SD16. Blood samples from all piglets were collected at 4‐, 7‐, 10‐, 14‐, and 21‐days postchallenge (DPC), and viral copy numbers were assessed by qPCR. As shown in Figure 7B, PRRSV genomic copy numbers in the Arbidol HCl‐treated group were lower obviously than those in the DMSO‐treated group. Additionally, proinflammatory cytokine levels in serum from different groups at 4, 7, 10, 14, and 21 DPC were detected by ELISA. As illustrated in Figure 7C, compared with the PRRSV/DMSO‐treated group, Arbidol HCl treatment significantly decreased the production of IL‐6, IL‐8, IL‐1β, and TNF‐α.

**Figure 7:**
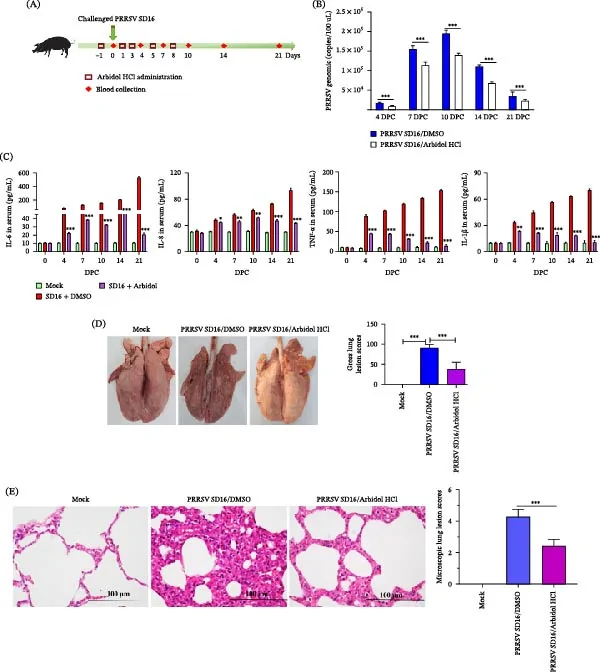
Protective effect of Arbidol HCl in PRRSV‐challenged piglets. (A) Outline of the piglet protection experiment. PRRSV‐free animals were allocated at random to three groups of five: PRRSV SD16/DMSO, PRRSV SD16/Arbidol HCl (40 mg/kg), and mock. (B) Serum viral copy numbers determined by qPCR at 4, 7, 10, 14, and 21 DPC, shown as mean ± standard deviation (error bars);  ^∗∗∗^ *p* < 0.001 against PRRSV SD16/DMSO piglets. (C) Serum IL‐6, IL‐8, IL‐1β, and TNF‐α at the same time points, measured with commercial ELISA kits;  ^∗^ *p* < 0.05,  ^∗∗^ *p* < 0.01 and  ^∗∗∗^ *p* < 0.001 against the PRRSV SD16/DMSO group. (D) Gross appearance and lesion scores of lungs from the three groups at 21 DPC. (E) H&E‐stained lung sections from each group (scale bar: 100 µm).

To further evaluate the therapeutic effect of Arbidol HCl against PRRSV‐induced lung injury, gross pathological examination was performed. The results demonstrated that lung gross lesions in the PRRSV SD16/Arbidol HCl group were significantly milder compared to the PRRSV SD16/DMSO group, which exhibited more severe pulmonary consolidation. In contrast, no pathological lesions were observed in the mock‐infected group (Figure 7D). Furthermore, microscopic examination revealed that lung lesions in PRRSV SD16/Arbidol HCl‐treated piglets were markedly less severe than those in the PRRSV SD16/DMSO group, which displayed thickened alveolar septa (Figure 7E).

## 3. Discussion

PRRS remains one of the most economically devastating diseases in the global swine industry, causing severe persistent infections, immunosuppression, and substantial financial losses [28]. Despite decades of research, effective therapeutic interventions remain limited; however, so far, no vaccines providing complete protection and no approved antiviral drugs are available for clinical use. This therapeutic gap underscores the urgent need for novel antiviral agents against PRRSV. In the present study, we demonstrate for the first time that Arbidol HCl, a broad‐spectrum antiviral agent, exhibits potent anti‐PRRSV activity through multiple complementary mechanisms: inhibition of viral entry via downregulation of cellular receptors CD163 and MYH9, suppression of virus‐induced inflammatory responses, and modulation of the TLR4/NF‐κB signaling pathway (Figure 8).

**Figure 8:**
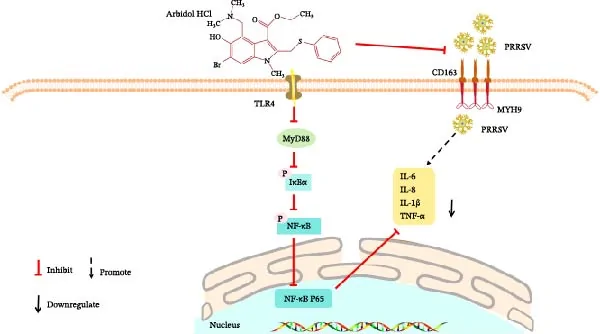
Proposed mechanism by which Arbidol HCl opposes PRRSV. By lowering CD163 and MYH9, Arbidol HCl prevents PRRSV from entering susceptible cells; at the same time, it limits release of the proinflammatory cytokines IL‐6, IL‐8, IL‐1β, and TNF‐α, in culture and in pigs alike, by damping the TLR4/NF‐κB cascade.

Arbidol HCl is a small‐molecule antiviral agent originally developed to combat influenza, with an established safety profile and a well‐characterized mechanism involving inhibition of viral membrane fusion and prevention of viral genome release [29]. Its broad‐spectrum activity has been documented against multiple viral families, including SARS‐CoV‐2, where it impedes viral attachment and endolysosomal release [30], and foot‐and‐mouth disease virus, where it disrupts genome replication [31]. Building on this evidence, we investigated whether Arbidol HCl could be repurposed as an anti‐PRRSV therapeutic. Our results demonstrate that Arbidol HCl exhibits dose‐dependent inhibition of PRRSV infection in both MARC‐145 cells and primary PAMs (Figure 2). Importantly, this antiviral activity extends across genetically diverse PRRSV isolates, including both PRRSV‐1 (GZ11‐G1 and P073‐3) and PRRSV‐2 (VR‐2332, JXA1, CH‐1a, and NADC30‐like) strains (Figure 3), suggesting that Arbidol HCl targets conserved features of the viral life cycle rather than strain‐specific elements.

Cellular receptors play a critical role in PRRSV infection, and CD163 and MYH9 were proved to be key mediators for PRRSV entry [32]. CD163 is a cysteine‐rich scavenger receptor on macrophages, which consists of nine tandem repeats of the SRCR domain (SRCR1 through SRCR9). It interacts with PRRSV GP2a and GP4 [33]. As a motor protein, MYH9 contributes to cell migration, adhesion, and morphogenesis, which interacts with the ectodomain of PRRSV GP5 during virus binding and internalization [34]. Drug treatments that modulate the CD163 or MYH9 expression of susceptible cells also affect PRRSV infection. For example, treatment with lipopolysaccharide (LPS) or tilmicosin can decrease the surface expression of CD163, which induces an inhibition of PRRSV infection [35, 36]. Additionally, treatment with specific siRNA or blebbistatin reduces the MYH9 expression of host cells, which inhibits viral infections of PRRSV [32, 37]. Our present study showed that Arbidol HCl could decrease CD163 and MYH9 expression (Figure 4K), which obviously reduces PRRSV infection.

A previous study reported that PRRSV infection triggers excessive generation of proinflammatory cytokines, which paradoxically enhances viral replication and exacerbates disease pathology [38, 39]. This inflammatory dysregulation represents a critical component of PRRSV pathogenesis. Previous studies have shown that Arbidol HCl possesses anti‐inflammatory properties in the context of influenza virus infection [40], but whether this extends to PRRSV‐induced inflammation remains unexplored. Our results demonstrate that Arbidol HCl significantly attenuates the production of key proinflammatory mediators, including IL‐6, IL‐8, IL‐1β, and TNF‐α, in both PRRSV‐infected PAMs and experimentally infected piglets (Figures 5 and 7). This dual antiviral and anti‐inflammatory activity suggests that Arbidol HCl may address both viral replication and immunopathology simultaneously.

To further dissect the potential molecular mechanisms of the anti‐inflammatory effects of Arbidol HCl, we employed targeted pathway validation. The TLR4/NF‐κB signaling cascade is pivotal for coordinating innate immune reactions and the synthesis of inflammatory cytokines [41]. Mechanistically, we demonstrated that Arbidol HCl dose‐dependently reduced the expression of TLR4 and MyD88 while decreasing phosphorylation of NF‐κB and IκBα, key events in pathway activation (Figure 6A). The specificity of this mechanism was confirmed through complementary approaches using TAK‐242 (a selective TLR4 inhibitor) and siRNA‐mediated TLR4 knockdown, both of which phenocopied the effects of Arbidol HCl on viral replication and cytokine production (Figure 6B–G). These findings establish that Arbidol HCl’s anti‐inflammatory and antiviral activities are mechanistically linked through suppression of the TLR4/NF‐κB signaling pathway.

The therapeutic potential of Arbidol HCl was further validated in vivo using a piglet challenge model. Arbidol HCl‐treated piglets exhibited significantly reduced viral loads in the blood throughout the course of infection and markedly decreased serum levels of proinflammatory cytokines compared to vehicle‐treated controls (Figure 7B,C). Importantly, Arbidol HCl treatment substantially ameliorated PRRSV‐induced lung pathology, with reduced gross lesions and less severe microscopic damage, including diminished alveolar septal thickening (Figure 7D,E). These data demonstrate that Arbidol HCl can confer protection against PRRSV infection in the natural host species.

In conclusion, this study provides comprehensive evidence that Arbidol HCl exerts potent anti‐PRRSV activity through multiple integrated mechanisms: (i) inhibition of viral entry by downregulating CD163 and MYH9 expression, (ii) suppression of virus‐induced inflammatory responses via the TLR4/NF‐κB pathway, and (iii) reduction of viral replication and associated pathology in vivo. The broad‐spectrum activity against diverse PRRSV strains, combined with its established safety profile in humans and other species, positions Arbidol HCl as a promising therapeutic candidate for PRRS management. Future studies should evaluate optimal dosing regimens, pharmacokinetic properties in swine, and efficacy in field conditions to facilitate translation to clinical application. This work not only identifies a potential new tool for PRRS control but also highlights the value of repurposing approved antiviral agents to address emerging and re‐emerging veterinary diseases.

## 4. Materials and Methods

### 4.1. Cells, Viruses, Reagents, and Antibodies

Two cell systems permissive for PRRSV were used throughout. MARC‐145 cells, sourced from the China Center for Type Culture Collection, were grown in Dulbecco’s modified Eagle medium (Gibco, Thermo Fisher, USA) made up with 10% fetal bovine serum (FBS, Lonsera, Uruguay) and 1% penicillin–streptomycin. PAMs were recovered by lung lavage from PRRSV‐free 8‐week‐old piglets following an established procedure [42] and maintained in RPMI‐1640 (Gibco, Thermo Scientific, USA) with the same serum and antibiotic supplements. Incubation was performed at 37°C under 5% CO_2_.

The seven PRRSV isolates used here were a gift from Dr. En‐Min Zhou (Northwest A&F University), including GZ11‐G1 (GenBank: KF001144.1) and P073−3 (GenBank: MK214314.1), which represent PRRSV‐1, whereas SD16 (GenBank: JX087437.1), VR‐2332 (GenBank: EF536003.1), CH‐1a (GenBank: AY032626), NADC30‐like (GenBank: KX766379), and JXA1 (GenBank: EF112445.1) belong to PRRSV‐2. Stock titers were established on MARC‐145 cells as the median tissue culture infective dose (TCID_50_) following an earlier report [43].

Arbidol HCl of greater than 99% purity was supplied by Shandong Sparkjade Biotechnology Co. Ltd., Jinan, China, reconstituted in DMSO at 10 mg/mL, and kept at −80°C until use. Antibodies recognizing IκBα, phospho‐IκBα, NF‐κB, phospho‐NF‐κB, TLR4, and β‐actin came from Servicebio, Wuhan, China, while monoclonal antibodies against the N protein of PRRSV‐1 and PRRSV‐2 were generated in our own laboratory. Secondary reagents, namely, HRP‐conjugated goat anti‐mouse and goat anti‐rabbit IgG and DyLight 594‐conjugated goat anti‐swine IgG, were obtained from Jackson ImmunoResearch (West Grove, PA, USA).

### 4.2. Cytotoxicity Detection Assay

Tolerance of the permissive cells to Arbidol HCl was measured with the cell counting kit‐8 (MedChemExpress, USA) according to the supplier’s instructions. Confluent monolayers of MARC‐145 cells or PAMs in 96‐well plates were exposed for 24 h at 37°C to 2.5, 5, 10, 20, or 40 µM of the compound, four wells per concentration alongside untreated wells. The monolayers were rinsed with PBS and then held for a further 2 h at 37°C in DMEM or RPMI‐1640 with 10% FBS and 10% CCK‐8 reagent. Absorbance at 450 nm was read on a microplate reader (BioTek, USA) and the 50% cytotoxic concentration (CC_50_) was derived in GraphPad Prism as described previously.

### 4.3. Determination of Antiviral Activity

The MARC‐145 cells, or PAMs, grown to roughly 90% confluence in 12‐well dishes, were held for 2 h in DMEM or RPMI‐1640 10% FBS containing 0, 5, 10, or 20 μM Arbidol HCl and then inoculated with PRRSV SD16 at 0.1 MOI for 1 h. The inoculum was exchanged for the corresponding medium containing 3% FBS and the same concentration of compound. Cells and culture fluids were harvested separately 24 hpi for further qPCR, western blot, and IFA.

### 4.4. Direct Inactivation Assay and Time‐Course Analysis

For the inactivation test, PRRSV SD16 at 0.1, 0.5, or 1 MOI was held with Arbidol HCl for 1 h at 37°C before the mixtures were applied to MARC‐145 cells for 1 h. Cells and supernatants taken at 24 hpi were examined by qPCR and western blot. Four regimens were then used to localize the drug‐sensitive step: compound present throughout (R1), before inoculation only (R2), during inoculation only (R3), or after adsorption only (R4). In each case, MARC‐145 cells received Arbidol HCl at 20 µM and PRRSV SD16 at 0.1 MOI as above, and material collected at 24 hpi was analyzed by qPCR and western blot.

### 4.5. Detection of Virus Attachment and Internalization

Attachment and entry were assayed essentially as reported previously. For attachment, MARC‐145 cells or PAMs plates were held with 20 µM Arbidol HCl or DMSO for 1 h at 37°C, transferred to ice, and exposed to PRRSV SD16 at 1 MOI for 1 h. Three washes in ice‐cold PBS removed unattached virions, and the bound fraction was quantified by qPCR against ORF‐7.

Entry was measured as follows: MARC‐145 cells or PAMs in 12‐well plates were preincubated with Arbidol HCl (20 µM) or DMSO for 1 h at 37°C and then held with PRRSV SD16 (1MOI) at 4°C for 1 h so that binding could occur without uptake. Unbound particles were washed away with chilled PBS, and the cells were shifted to 37°C for 1 h in 3% FBS DMEM containing 20 µM Arbidol HCl or DMSO to permit internalization. Virions remaining on the surface were stripped with citrate buffer at pH 3.0, and internalized genomes were enumerated by qPCR.

### 4.6. Real‐Time qPCR Assay

Procedures followed those described previously [44, 45]. Cells harvested at the specified intervals were extracted with the SparkZol Reagent (Sparkjade, Jinan, China) as directed by the supplier. First‐strand synthesis used PrimeScript RT Master Mix (Takara, Dalian, China), and amplification was performed with 2×RealStar Green Fast Mixture (GenStar, China) together with the primers listed in Table 1. GAPDH served to normalize the input, and relative abundance was derived by the 2^−∆∆Ct^ method. Absolute genome copy numbers of PRRSV were obtained in parallel against a standard curve built from the recombinant plasmid pMD18‐T‐N, which carries the PRRSV ORF‐7 gene.

**Table 1:** List of primers used in this study.

Genes	Forward primer (5′–3′)	Reverse primer (5′–3′)
^a^ *ORF7*	ATGGCCGGTAAAAATCAGAGCC	TTAATTCGCACCCTGACTGG
^b^ *ORF7*	ATGCCAAATAACAACGGCAAGC	TCATGCTGAGGGTGATGCTGTG
*siTLR4*	GCAAATGCCTCTGTGATTT	GCAAATGCCTCTGTGATTT
*pig-IL-6*	CAGCCCTGAGAAAGGAGACA	CCAGGCAAGTCTCCTCATTG
*pig-IL-8*	ACTCCAAACCTATCCACCCC	CCACAACCCTAGACACCCAT
*pig-TNF-α*	TCCCCAGGAAGACAGCG	GCAGCAGACAGAAGAGCGT
*PAM-IL-1β*	ACCTGGACCTTGGTTCTCTG	CATCTGCCTGATGCTCTTGT
*MYH9*	GACCAGATCAACACCGACCT	ACTTGACTGTGCCCTCCATC
*CD163*	TTCACTGCACTGGGACTGAG	AGGACAGTGTTTGGGACTGG
*GAPDH*	GTCTCCTCTGACTTCAACAGCG	ACCACCCTGTTGCTGTAGCCAA

^a^Used for PRRSV‐1.

^b^Used for PRRSV‐2.

### 4.7. Western Blot Analysis

Immunoblotting followed a published protocol [46]. Cells taken at the indicated intervals were disrupted in NP40 lysis buffer (Beyotime, Nanjing, China) and cleared by centrifugation, and the protein in the supernatant was quantified with the Pierce BCA Protein Assay Kit (Thermo Scientific, USA). Lysates combined with 5×SDS protein loading buffer were resolved on 10% SDS‐PAGE gels and transferred to polyvinylidene difluoride (PVDF) membranes. Membranes were incubated with the relevant primary antibody and then with HRP‐conjugated goat anti‐mouse antibody or goat anti‐rabbit IgG. The signal was developed with an ECL substrate (Thermo Fisher, USA) and recorded on a ChemiDoc MP Imaging System (BioRad, USA).

### 4.8. IFA

Staining was carried out as reported earlier [47]. Briefly, MARC‐145 cells or PAMs grown on coverslips in 12‐well plates were exposed to 5, 10, or 20 µM Arbidol HCl for 2 h and then to SD16 at an MOI of 0.1. At 48 hpi, the monolayers were fixed in ice‐cold 75% ethanol, incubated with porcine PRRSV convalescent serum raised in our laboratory, and then with DyLight 594‐conjugated AffiniPure goat anti‐swine IgG (H + L) (Jackson, PA, USA). Nuclei were counterstained with DAPI (Beyotime, China), and fields were recorded on a Leica DMi8 fluorescence microscope (Germany).

### 4.9. Virus Titration Detection

Infectious titers were determined on MARC‐145 cells by the Reed–Muench approach [48] following a published protocol. Monolayers at 80%–90% confluence in 96‐well cell plates received 100 μL of 10‐fold serial dilutions of each supernatant, eight wells per dilution. One hour after inoculation, the medium was exchanged for DMEM with 3% FBS, and the cytopathic effect was scored 5 days postinfection (dpi). The 50% tissue culture infective dose (TCID_50_) was computed in GraphPad Prism 8.0.

### 4.10. Cytokine Detection by ELISA

PAMs were exposed to PRRSV SD16 at an MOI of 0.1 with or without 10 or 20 μM Arbidol HCl, and culture fluids collected at 24 hpi were assayed for IL‐6, IL‐8, IL‐1β, and TNF‐α using commercial ELISA kits (Jianglai, China) as directed by the manufacturer. Sera taken from the mock, PRRSV SD16/DMSO, and PRRSV SD16/Arbidol HCl groups at 4, 7, 10, 14, and 21 DPC were tested for the same four cytokines with the identical kits.

### 4.11. siRNA Knockdown Assay

siRNA directed against TLR4 and a nontargeting control (siNC) were synthesized by Gene Pharma Co., Ltd.; the sequences appear in Table 1. PAMs seeded in 12‐well plates overnight were transfected with 100 nM siTLR4 or siNC using Lipo6000 (Beyotime, China) for 10 h and then held with 20 μM Arbidol HCl for 2 h. Infection with PRRSV SD16 at 0.1 MOI proceeded for 1 h, after which the medium was exchanged for RPMI‐1640 with 3% FBS and the cultures maintained for a further 24 h. RNA extracted with SparkZol Reagent (SparkJade, China) served for RT‐qPCR quantification of IL‐6, IL‐8, IL‐1β, TNF‐α, and ORF7 by the 2^−ΔΔCT^ method with GAPDH as reference. Parallel lysates were immunoblotted for PRRSV N protein, TLR4, MyD88, p‐NF‐κB, NF‐κB, p‐IκBα, and IκBα.

### 4.12. In Vivo Anti‐PRRSV Activity of Arbidol HCl Detection and Hematoxylin and Eosin (H&E) Detection

Fifteen PRRSV‐free pigs aged 8 weeks, obtained from Pengyue Laboratory Animal Technology Co., Ltd. (Jinan, China), were assigned at random to three groups of five. Animals in group 1 were inoculated intranasally with SD16 at 3 × 10^5^ TCID_50_ per piglet. Group 2 received the same challenge together with Arbidol HCl at 40 mg/kg. Group 3 served as the mock group and was given DMSO alone. Arbidol HCl was administered orally to group 2 on days−1, 1, 3, 5, and 8 relative to challenge (DPC), while groups 1 and 3 were dosed orally with an equal volume of MEM containing 0.1% DMSO. Blood was sampled at 4, 7, 10, 14, and 21 DPC for quantification of viral load and proinflammatory mediators. At 21 DPC, every animal was deeply anesthetized and then killed by intravenous sodium pentobarbital (100 mg/kg, via ear vein) at no less than three times the anesthetic dose, which produced rapid loss of consciousness followed by cardiopulmonary arrest; death was verified by the absence of heartbeat, respiration, and corneal reflex. Lungs were examined grossly and retained for histology. Tissue was fixed in 10% formalin, dehydrated, embedded in paraffin, cut at 4 µm, and stained with H&E. Macroscopic involvement was recorded as affected, and microscopic change was graded on a 4‐point scale: 0, no lesion; 1, mild focal or multifocal interstitial pneumonia involving less than 50% of the tissue; 2, moderate multifocal to coalescing lesions covering 50%–75%; 3, severe patchy, coalescing, and widespread lesions affecting 75%–90%; and 4, severe diffuse lesions extending beyond 90%.

### 4.13. Statistical Analysis

Analyses were carried out in GraphPad Prism 8.0. Group comparisons used an unpaired Student’s *t*‐test or one‐way ANOVA as appropriate. Asterisks denote  ^∗^, *p* < 0.05;  ^∗∗^, *p* < 0.01;  ^∗∗∗^, *p* < 0.001.

## Author Contributions

**Li Chen and Mengxue Gao**: conceptualization, data curation, formal analysis, investigation, methodology, validation, visualization, writing – original draft, writing – review and editing. **Xiaoyang Zhan** **, Lei Kong, Shuzhen Jiang, Suimin Shang, En-xin Li, Xiaoting Peng, Qiuju Dai, Yubao Li, Yuyu Zhang**, **and Muhammad Zahoor Khan**: data curation, formal analysis, investigation, methodology, writing – review and editing. **Jiaqiang Wu and Tongtong Wang**: writing – review and editing, writing – review and editing, supervision, validation. **Liangliang Li, Jiaqiang Wu, and Tongtong Wang**: conceptualization, funding acquisition, validation, methodology, project administration, resources, software, supervision, writing – original draft, writing – review and editing.

## Funding

The present research was financially supported by the China Postdoctoral Science Foundation (Grant 2025M773037, recipient: Tongtong Wang), the Shandong Provincial Agricultural Major Technology Collaborative Promotion Program (Grant SDNYXTTG‐2025‐43), the Natural Science Foundation of Shandong Province (Grants ZR2024MC162 and ZR2026MS0410), the Shandong Province Pig Industrial Technology System (Grant SDAIT‐08‐01, recipient: Jiaqiang Wu), and the Youth Project of Shandong Province Higher Educational Science and Technology Program (Grant 2022KJ287, recipient: Tongtong Wang).

## Disclosure

All authors have read and approved the published version of the manuscript.

## Ethics Statement

All experimental protocols were approved by the Liaocheng University Animal Care and Use Committee (Permit Number AP2025092902) and were performed according to the Animal Ethics Procedures and Guidelines of the AVMA. No other specific permissions were required for these experiments. This study did not involve any endangered or protected species.

## Conflicts of Interest

The authors declare no conflicts of interest.

## Data Availability

The original data obtained in the study are included in the article. Further inquiries can be directed to the corresponding authors.
